# Correction: Engineering of antimicrobial peptide fibrils with feedback degradation of bacterial-secreted enzymes

**DOI:** 10.1039/d6sc90167k

**Published:** 2026-08-18

**Authors:** Fenghua Wang, Wencheng Xia, Mingming Zhang, Rongrong Wu, Xiaolu Song, Yun Hao, Yonghai Feng, Liwei Zhang, Dan Li, Wenyan Kang, Cong Liu, Lei Liu

**Affiliations:** a Institute for Advanced Materials, School of Materials Science and Engineering, Jiangsu University Zhenjiang Jiangsu 212013 China liul@ujs.edu.cn; b School of Aeronautical Engineering, Jiangsu Aviation Technical College Zhenjiang Jiangsu 212134 China; c Interdisciplinary Research Center on Biology and Chemistry, Shanghai Institute of Organic Chemistry, Chinese Academy of Sciences Shanghai 201210 China liulab@sioc.ac.cn; d Bio-X Institutes, Key Laboratory for the Genetics of Developmental and Neuropsychiatric Disorders (Ministry of Education), Shanghai Jiao Tong University Shanghai 200030 China; e Zhangjiang Institute for Advanced Study, Shanghai Jiao Tong University Shanghai 200240 China; f Department of Neurology and Institute of Neurology, Ruijin Hospital, Shanghai Jiao Tong University School of Medicine Shanghai 200025 China; g Department of Neurology, Ruijin Hainan Hospital, Shanghai Jiao Tong University School of Medicine (Boao Research Hospital) Hainan 571434 China; h State Key Laboratory of Bio-Organic and Natural Products Chemistry, Shanghai Institute of Organic Chemistry, University of Chinese Academy of Sciences Shanghai 200032 China

## Abstract

Correction for ‘Engineering of antimicrobial peptide fibrils with feedback degradation of bacterial-secreted enzymes’ by Fenghua Wang *et al.*, *Chem. Sci.*, 2023, **14**, 10914–10924, https://doi.org/10.1039/d3sc01089a.

The authors regret that the affiliation details listed for Fenghua Wang were incorrect in the original article. The correct list of affiliations for this article is as shown herein.

The Royal Society of Chemistry apologises for these errors and any consequent inconvenience to authors and readers.

